# Perceptions of environmental health risks among residents in the “Toxic Doughnut”: opportunities for risk screening and community mobilization

**DOI:** 10.1186/s12889-015-2563-y

**Published:** 2015-12-10

**Authors:** Brandi M. White, Eric S. Hall

**Affiliations:** Department of Health Professions, College of Health Professions, Medical University of South Carolina, 151 Rutledge Avenue, Charleston, SC 29425-1600 USA; Office of Research and Development, US Environmental Protection Agency, 109 T.W. Alexander Drive, Research Triangle Park, NC 27711-0001 USA

**Keywords:** Environmental justice, Risk perceptions, Community assessment

## Abstract

**Background:**

Surrounded by landfills, and toxic and hazardous facilities, Altgeld Gardens is located in a “toxic doughnut”. With high rates of environmentally-related conditions, residents have called for a community-based environmental health assessment to improve overall health in their community. The purpose of this study was to investigate the attitudes and beliefs of environmental health risks of Altgeld’s residents which would assist community organizing efforts and provide the groundwork for a community-based environmental health assessment.

**Methods:**

A questionnaire was designed and administered to 42 Altgeld residents who also participated in focus groups to assess their perceptions of environmental health risks.

**Results:**

All participants were Altgeld residents for at least two years and were fairly representative of the broader community. Physical and social hazards were primarily identified as posing risks to participants’ family and the broader community. Physical hazards included the dumping of hazardous waste and landfills; social hazards were crime and drugs.

**Conclusions:**

These findings have been useful in community organizing efforts and in program planning for local community-based organizations and public health agencies. The results have also been used to prioritize health and environmental risk issues impacting the community.

**Electronic supplementary material:**

The online version of this article (doi:10.1186/s12889-015-2563-y) contains supplementary material, which is available to authorized users.

## Background

**Fig. 1:**
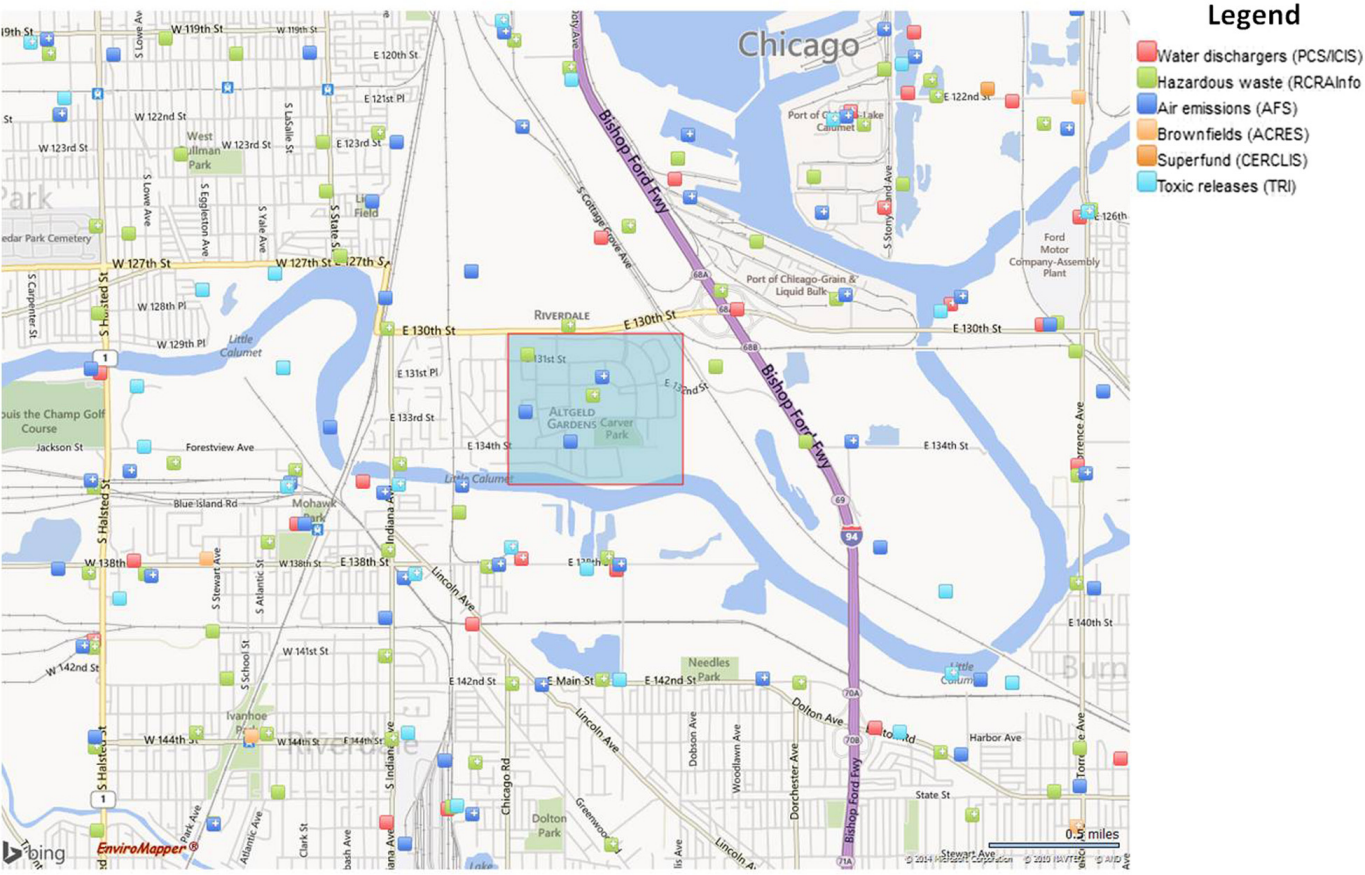
Altgeld Gardens and Surrounding Area

Few communities are surrounded by so many hazardous facilities that they are coined to be in a “toxic doughnut” (see Fig. 1). Altgeld Gardens and Phillip Murray Homes (herein referred to as ‘Altgeld’) is one community that is referred to as such. A public housing development with predominately black residents in the Calumet industrial area in Southeast Chicago, Illinois, Altgeld has a long history of environmental activism through the efforts of People for Community Recovery (PCR), a resident-led social justice organization in the community. Originally built to house black veterans after World War II, Altgeld was one of the first public housing developments in the United States. The development was built on an abandoned waste site and is surrounded by the most landfills per square mile in the United States [1]. Residents have voiced their concerns for several years, including concerns regarding soil contamination which is used by many for local gardening [2]. In 2011, facilities in the area released over 3.5 million pounds of toxic waste, accounting for almost 30 % of all toxic releases in Cook County, Illinois [3].

In addition to the large quantities of hazardous materials released in the area and the resulting potential for population exposures to these materials, the community has high rates of illness, many of which are associated with toxic exposures in the physical environment. The area has some of the highest mortality rates for lung cancer and cerebrovascular disease (stroke) in the city [4]. In previous community health surveys, residents have identified concerns about environmental risk factors for lung cancer, such as asbestos, environmental tobacco smoke, and radon. For stroke, proximity to hazardous facilities and exposure to air pollution are both strong contributors to incidence and mortality rates [5]. In addition to high rates of environmentally-related diseases, the community has the highest percentage of people living in poverty and the lowest per capita income in the city [4].

Thus, this community meets the two criteria defining an environmental justice (EJ) community. First, the community has experienced historical (usually multi-generational) exposures to disproportionately high doses of potentially harmful substances (the *environmental* part of the definition) [6–9]. Altgeld is home to numerous pollution sources, including heavy industry and pollution control facilities, which may be obvious by their stacks and outfall structures, or which may be more subtle, such as long buried wastes with little evidence on the surface of their existence. These sites increase the likelihood of exposure to dangerous substances. Exposure is preferred to *risk*, since risk is a function of the hazard and the exposure to that hazard. Even a substance with a very high toxicity (one type of hazard) that is confined to a laboratory of a manufacturing operation may not pose much of a risk due to the potentially low levels of exposure.

Second, EJ communities have a majority representation of low socioeconomic status, racial/ethnic minorities and/or historically disadvantaged people (the *justice* part of the definition) [10–12]. Altgeld is a public housing development, indicating that this is an economically disadvantaged group, which poses several other public health concerns, i.e. access to healthy food and adequate medical services. Furthermore, it is a predominately African American community (see Table 1), which historically have been disproportionately exposed to environmental hazards [12].

**Table 1:** Demographic characteristics of study participants and the larger community

Characteristic	Study participants		Riverdale community		*P*-value
	Numerical value	Percentage	Numerical value	Percentage	
Total	42	**-**	9,809	**-**	
Age (years)					
Median	49.0	**-**	20.5	**-**	
Average	45.1 (±13.5)	**-**	NA	**-**	
Gender					
Female	26	61.9 %	5,524	56.5 %	0.4799
Male	16	38.1 %	4,285	43.5 %	
Race/Ethnicity					
Black/African American	41	97.7 %	9,495	96.8 %	0.7630
Hispanic	1	2.3 %	314	3.2 %	
Education					
HS Grad	23	54.8 %	6,121	62.4 %	0.3068
Not HS Grad	19	45.2 %	3,688	37.6 %	
Marital Status					
Never Married	24	57.1 %	3,266	33.3 %	0.1888
Married	18	42.9 %	6,543	66.7 %	
Employment					
Unemployed	15	35.7 %	3,286	33.5 %	<0.05
Employed	27	64.3 %	6,523	66.5 %	
Health Status					
Fair or Poor	18	42.9 %	6,543	66.7 %	<0.05
Better than Fair or Poor	24	57.1 %	3,266	33.3 %	

Source: Data on the Riverdale Community is from the Illinois Project for Local Assessment of Needs, 2000; *NA* Not available, *HS* High School

**Table 2:** Percentage of persons agreeing or disagreeing with survey statements by category (*N* = 42)

Statement	Strongly agree	Agree	Disagree	Strongly disagree
*Government Agencies*				
I believe there are enough laws to control environmental risks.	14.3	26.2	28.6	31.0
When there is a really serious health problem, the government will do something about it. Until they tell me about a specific problem, I don’t have to worry.	11.9	23.8	33.3	30.9
*Environmental Solutions*				
If people work together, they can change the environment.	64.3	21.4	2.4	11.9
There are things I can do that will make a difference in improving the environment.	57.1	26.2	11.9	4.8
Nothing can be done about environmental problems like hazardous waste and air pollution.	14.3	16.7	11.9	57.1
*Environment and Health Risks*				
Most chemicals cause cancer.	47.6	33.3	11.9	7.1
The risk of getting cancer from things like smoking and diet is much greater than the risk of cancer from chemicals in the environment.	33.3	21.4	16.7	28.6
People can protect themselves against health risks from pollution by improving their individual lifestyle, such as by exercising and eating properly.	33.3	21.4	16.7	28.6
If even a tiny amount of a substance that could make me sick were found in my tap water, I wouldn’t drink it.	45.2	11.9	23.8	19.0
I don’t worry about chemicals because there are just too many other things in my life I have to deal with.	7.1	21.4	19	52.4
I feel I have very little control over risks to my health.	11.9	26.2	14.3	47.6

**Table 3:** Percentage of respondents indicating that hazard is “high risk/very high risk to myself and my family”, and “high risk/very high risk to community as a whole” (*N* = 42)

Very high/High risk hazard	Community as a whole	Self & family	Difference
Crime	79	60	19
Drugs	79	52	27
Dumping hazardous waste	74	69	5
Landfills	74	62	12
Chemicals	69	64	5
Smoking	69	52	17
Lead	69	55	14
Outdoor air pollution	67	60	7
HIV/AIDS	64	45	19
Police brutality	60	45	15
Sewage	57	50	7
Waste incinerators	57	38	19
Depletion of ozone layer	52	36	16
Asbestos	52	36	16
Pesticides in food	52	33	19
High tension wires	45	43	2
Global warming	48	33	15
Indoor air pollution	45	33	12
Drinking water	43	26	17
Bacteria in food	38	33	5
Car accidents	36	24	12
Sun exposure	33	21	12

Note: The difference was obtained by subtracting the percentage answering “very high risk/high risk” for each hazard for self from that for the community. The hazards posing the highest risk and lowest risk are in bold text

**Table 4:** Percentage of environmental information received from sources by degree (*N* = 42)

Source	A lot	A fair amount	A little	Almost none
Chicago City Health Department	9.5	21.4	33.3	35.7
Chicago Housing Authority	9.5	14.3	23.8	52.4
Doctor	19.1	14.3	21.4	45.2
Friends and relatives	31.0	14.3	28.6	26.2
Illinois Department of Health	16.7	19.1	21.4	42.9
Newspaper	21.4	21.4	40.5	16.7
People for Community Recovery (PCR)	45.2	21.4	21.4	11.9
Private industry, such as Waste Management or Ford Motor Company	9.5	7.1	19.1	64.3
Radio	21.4	14.3	21.4	42.9
Television	31.0	28.6	33.3	7.1
U.S. Environmental Protection Agency	-	40.5	28.6	30.9
University/College scientists	4.8	26.2	33.3	35.7

Understanding residents’ perceptions of environmental hazards, especially those that are identified as environmental justice communities, is also essential [13]. Faith and trust in health agencies and the communication of environmental risks by these agencies influence individual perceptions [14, 15]. Race and socioeconomic status also influence perceptions of hazards, especially for African Americans [14, 16]. Physical alienation, such as residential segregation, and perceived power among are also powerful determinants of perceptions of hazards among African Americans [14, 17].

Because of the multiple environmental, health, and socioeconomic concerns of the Altgeld community, residents and community leaders have recommended conducting a community-based environmental health assessment to inform recommended improvements to environmental conditions in the community, mitigate toxic exposures, and improve health. A community-based environmental health assessment is a strategy to engage community members in improving the physical environment and overall community health [18].

The assessment involves identifying community-specific environmental health concerns; prioritizing concerns; empowering community residents to organize and participate in community cleanups; and implementing strategies to mitigate and/or prevent toxic exposures [19]. Findings from an assessment are especially important for decision-making and resource allocation [20, 21]. Therefore, the purpose of this study was to investigate the attitudes and beliefs of environmental health risks of Altgeld’s residents which would assist community organizing efforts and provide the groundwork for a community-based environmental health assessment.

## Methods

The research protocol for this study was approved and monitored by the Institutional Review Board of the University of Minnesota (IRB Study Number: 0812P55541). PCR provided guidance on with the questionnaire, assisted with recruitment, and helped to interpret the findings. The U.S. EPA’s Office of Research and Development (ORD) provided expertise for the post-study analysis of the data and results. ORD reviewed the statistical summary of the questionnaire results and did not have access to personally identifiable information, individual responses, or individual demographic information.

### Data collection

This study used a non-probability sample because of convenience and cost. Data collection occurred in March 2009. Participants were recruited using flyers and advertisements at PCR’s office, the local convenience store, and the neighborhood health clinic within one week of data collection. To be eligible, participants had to be between the ages of 18 and 64, and a permanent resident of Altgeld for at least two consecutive years. The primary data collection method for the study was qualitative (focus groups); these findings are reported elsewhere [2]. Modified questionnaires were administered prior to the focus group. The focus group moderator provided assistance to participants needing support completing the survey. Findings from the focus groups were validated with a follow-up survey with a separate group of residents (respondent validation survey). This ensured that the beliefs of the first group were representative of the community. All participants were compensated for their time. Verbal informed consent was obtained from research participants prior to completion of the questionnaire and start of the focus group. Written consent was obtained from participants who completed the respondent validation survey.

### Questionnaire

A close-ended questionnaire collecting information on attitudes and beliefs about environmental health risks and environmental information was modified from a previous questionnaire with input from PCR (see Additional file 1) [13, 22]. The questionnaire has not been validated, but it has been used with other low-income, minority communities [13, 22]. This questionnaire was pilot tested with Altgeld residents who did not participate as research participants to ensure it was appropriate. The questionnaire included items regarding attitudes about environmental regulations and experts, perceptions of risks from various environmental hazards, and attitudes and beliefs about how to address environmental problems. [13, 22] To assess these attitudes and beliefs, participants were asked to read a series of statements and to agree or disagree with each one, using a four-point Likert scale ranging from “strongly agree” to “strongly disagree”.

The questionnaire also included items regarding sources of environmental information. To assess general perceptions of risk, participants were given a list of hazards, and asked to rate the “riskiness” of each item, both for themselves and for the community as a whole, using a five-point Likert scale ranging from “very high risk” to “almost no risk”. Hazard items included physical hazards (e.g. waste incinerators), hazards related to risky health behavior (e.g., HIV/AIDS), and hazards linked to consumer goods (e.g., car accidents). Demographic information included age, gender, marital status, race/ethnicity, and education.

### Data analysis

Descriptive statistics were generated for the questionnaire using SAS Software (Version 9.2., Cary, NC). Means, medians, standard deviations, and ranges were used to describe continuous variables including age. Frequencies were used to describe categorical variables such as those in the questionnaire. Chi-square tests were used to determine if the observed demographic characteristics of participants were significantly different from that of the expected community. The level of statistical significance was *p* < 0.05.

## Results

### Study participants

All 42 participants who completed a questionnaire were current residents of Altgeld. Data collection for the focus groups and questionnaire ended once saturation was met with the focus group discussions. The mean age of participants was 45.1 (SD ± 13.5) and more than half of participants were female (62 %). All of the participants were racial/ethnic minorities (African American: 97.7 % and Hispanic: 2.3 %). Most were high school graduates (79 %) and over one third were unemployed (36 %). When asked to describe their general health, almost half reported their health as fair or poor (43 %). Table 1 shows demographic information about the sample in this study in comparison to residents in the community. In comparison to the larger community, participants in this study were more likely to be employed and differed in self-reported health status.

### Attitudes and beliefs

In general, beliefs towards government agencies were negative (see Table 2). More than half of the participants believed there were not enough laws to regulate environmental health risks (60 %). In contrast, most believed in the potential for solving environmental problems (86 %) and that people working together can improve the environment (83 %). In addition, while a majority of the participants somewhat or strongly agreed that “most chemicals cause cancer” (81 %), more than half believed that risks posed by smoking and diet were greater than risks due to chemicals in the environment (55 %).

### Risk perceptions

The dumping of hazardous waste, chemicals, and landfills were seen as posing the highest risks to participants and their families respectively at 69 %, 64 %, and 62 % (see Table 3). Drinking water, car accidents, and sun exposure were seen as posing the least risk to individuals (26 %, 24 %, and 21 % respectively). Crime, drugs, landfills, and dumping hazardous waste were seen as the highest risks to the community (79 %, 79 %, 74 %, and 74 % respectively). Conversely, sun exposure, car accidents, and bacteria in food were seen as posing the least risk to the community (33 %, 36 %, and 38 % respectively). Risks to the broader community were ranked higher than risks posed to the participant and his/her family.

### Source of environmental information

Participants reported getting “a fair amount” to “a lot” of information about the environment from PCR (67 %), the television (60 %), and friends and relatives (45 %) (see Table 4). Public agencies were not a major source of information. Forty percent (41 %) received “a fair amount” of information from the U.S. EPA. From the Illinois Department of Health, 36 % reported receiving “a fair amount” to “a lot” of information; 31 % from the Chicago City Health Department; and 24 % from the Chicago Housing Authority. Participants received the least amount of information from private industry (almost no information: 64 %).

## Discussion

The purpose of this study was to investigate the attitudes and beliefs of environmental health risks of a community surrounded by environmental hazards in order to improve local community organizing efforts and provide the foundation for a community-based environmental health assessment. Findings from assessment can be used to design health education campaigns, prioritize environmental health hazards for remediation, and be catalyst for social justice. Our sample reported that they did not feel adequately protected from environmental hazards; however, they had confidence that their community could work together to improve their environment. Study participants identified several physical and social hazards as risks to their family and the larger community; the dumping of hazardous waste, landfills, crime, and drugs were the most hazardous risks cited. In addition, participants indicated that they received a large amount of environmental information from PCR, the community-based organization serving the community.

The methodology employed in this study and its findings are important for several reasons. ***First***, this study engaged PCR, a resident-led, community-based organization, and Altgeld residents in the identification of community-specific environmental health concerns. This approach used a grassroots approach by partnering with PCR in the study design, questionnaire development, and data analysis. These efforts allowed for the community most impacted by environmental hazards to be engaged in the research process and engage with research scientists, thus providing a more realistic view of the community’s environmental conditions. This approach demonstrates that residents can contribute in developing intervention strategies focused on mitigating the impact of environmental hazards in their communities and in reducing the number of their environmental health concerns. Furthermore, it is essential to tailoring intervention strategies and programs for communicating clear and relevant environmental health messages.

Previous studies have involved community members in the identification of environmental health issues by engaging residents in the research process and acknowledging the community’s concerns [23, 24]. One such approach, community-based participatory research (CBPR), has been used to create and inform environmental health education campaigns to communicate environmental health risks. CBPR is a collaborative approach to research that engages community and university partners in all research phases. One CBPR partnership developed a community-specific environmental health campaign for low-income minority communities with environmental justice concerns [23]. By tailoring environmental health education material for these communities, the partnership was able to effectively communicate risks, enhance environmental health literacy, and support community organizing efforts around environmental justice. Thus, community engagement and community-based environmental health assessments are critical to improve overall environmental conditions and community health outcomes.

***Second,*** the survey allowed PCR to identify community-specific risks and concerns; thus, it acted as an instrument for community organizations and leaders to use when identifying environmental health concerns. Community-based environmental health assessments begin by noting and understanding perceptions of community-specific environmental health issues [18, 25]. The survey provided a list of environmental health risks and issues that PCR identified and participants confirmed as relevant. The risks were limited to those identified by PCR and the existing questionnaire, making data interpretation for community organizing efforts more manageable. This process is similar to the suggested tasks in the Protocol for Assessing Community Excellence in Environmental Health (PACE EH), developed by the National Association of County and City Health Officials and the Centers for Disease Control and Prevention [25]. PACE EH provides guidance for community organizations and health agencies conducting environmental health assessments by outlining thirteen essential tasks to improve community health.

In addition, the information obtained from the questionnaire could be used in conjunction with environmental justice tools such as the Environmental Justice Screening Methodology (EJSM), developed for use in EPA Region 9 [26]. Use of the EJSM tool requires validation (“ground-truthing”) of the data used to assess environmental hazards in the community. The data validation activity for EJSM is implemented by community members who are familiar with environmental hazards in the community. Knowledge of the local environmental hazards is a necessary prerequisite to data validation for EJSM. The questionnaire findings indicate that Altgeld residents are extremely aware of the local environmental hazards and would be capable of validating and finding additional environmental hazards if the EJSM tool, or one similar to it, is applied in their area.

***Third***, the survey obtained information on the components of the Health Belief Model (HBM) to assist in improving community participation in local organizing efforts. The HBM is a health behavior change model commonly used in community health promotion efforts that weigh the risk and benefits of action [27]. The HBM consists of the perceived susceptibility and severity of a health issue, and the perceived benefits of changing a behavior to protect one’s health [27]. The survey obtained information on participant’s perceived susceptibility to environmental hazards; residents believed they were not protected by hazards that could potentially cause cancer. These hazards were especially severe, posing high risks to themselves, their family, and their community based on the survey’s rank of a hazard’s riskiness (perceived severity). Because of the risks posed by the hazards, participants believed their community could work together to change their environment (perceived benefit), and more specifically that they could make a difference.

Together, the information for each HBM component can assist with developing educational material to mobilize residents to become involved in community organizing efforts. More specifically, educational material would focus on the adverse health impacts of community-specific hazards to residents’ families and the broader community. For example, communities that live in close proximity to municipal landfills have poor quality air and residents are at an increased risk for respiratory symptoms and decreased activities because of severe, bad odors [28]. Residents armed with this information who experience high rates of respiratory infections and are exposed daily to bad odors would challenge local legislation to prevent the further expansion of existing landfills.

***Fourth***, after obtaining information about perceptions of environmental hazards, the survey provided an opportunity for participants to rank a select group of community-specific risks. Participants ranked environmental health risks by describing how hazardous the risks were to them, their family, and their community on a Likert scale. Social hazards were ranked highest from the perspective of the participant and his/her family, and physical hazards were ranked the highest from the perspective of the community. This is not surprising as participants most likely saw social hazards as an immediate threat to the well-being of themselves and their families.

For community organizations leading community-based environmental health assessments, such as PCR, this information is especially important when engaging key stakeholders in removing or remediating hazards. For instance, participants identified the dumping of hazardous waste as a high risk to their community. As any dumping of waste in a residential area is illegal, PCR and residents were able to document the location and provide images to regulatory agencies for monitoring and prosecution of violators. Most recently, PCR, Altgeld residents, and other environmental groups organized to protest the expansion of an existing landfill that borders the community. Their organized efforts convinced the governor to sign legislation to ban any expansion of the landfill.

***Fifth***, the information obtained from the survey has helped considerably in program planning for PCR. PCR is one of the founding members of the Environmental Justice Alliance of the Greater Southeast Chicago, a coalition of local organizations dedicated to improving the environment and health of residents on the Southeast side of Chicago. The information from the questionnaires has been used to determine which areas the community wants to target for investigation and enforcement. The coalition has obtained support from larger environmental groups such as the Sierra Club, which shares common objectives, as well as local businesses that want to invest in the community.

In addition to PCR and other local organizations, the information from the questionnaire can be used by public health agencies and clinics to plan health and wellness campaigns that demonstrate what residents can do with their health and lifestyle choices to mitigate the impact of environmental hazards. Based on the questionnaire, participants understood the importance of lifestyle factors in health; however, most received little to no information from their healthcare provider or local public health agencies. The survey provides an opportunity for providers and agencies to target their resources and deliver community-specific health education material. The information can also be used by local, state, and regional planning agencies to ensure that as new housing, industrial, infrastructure, and other developments are implemented, that community input is included to ensure that there are employment opportunities, ‘green spaces,’ and urban planning designs that facilitate healthy lifestyles and sustainable communities. In addition, the findings provide an opportunity to apply a more comprehensive tool, such as the EJSM, to further examine local environmental conditions.

What is especially unique about this study is that the findings have been used to engage local residents, assist in community organizing efforts, and inform future directions to improve local conditions in collaborative partnerships. The detailed findings from this study have been used to support PCR’s community organizing efforts, and there are opportunities for continued use of the study information in community education and local program planning. Some of the limitations of this study are noted here. These findings cannot be generalized across all African-American public housing residents living near environmental hazards or the residents of Altgeld Gardens because a convenient sample was recruited. The list of environmental health risks were limited to those identified by PCR and the existing questionnaire. Despite the limitations, the findings provide important informative data regarding what can be done to effectively communicate environmental risks to low-income, African-American communities.

## Conclusion

This study demonstrated that a community-based questionnaire can inform community educational efforts and assist in local organizing efforts. This study used a community-engaged research approach to address environmental health concerns among public housing residents. These methods and findings can be used to inform program planning for community-based organizations and other public health agencies.

## Additional file

Additional file 1:
**Focus group survey.** (DOCX 23 kb)

## Abbreviations

CBPR: Community-based participatory research
EJ: Environmental justice
EJSM: Environmental Justice Screening Methodology
HBM: Health Belief Model
ORD: Office of Research & Development
PCR: People for Community Recovery
PACE EH: Protocol for Assessing Community Excellence in Environmental Health
U.S. EPA: U.S. Environmental Protection Agenc
