# Supplementary material for: Perceptions of environmental health risks among residents in the “Toxic Doughnut”: opportunities for risk screening and community mobilization
Source: BMC Public Health. 2015 Dec 10;15:1230. doi: 10.1186/s12889-015-2563-y (PMC4676177; doi:10.1186/s12889-015-2563-y)
Supplement: Additional file 1: — Focus group survey. (DOCX 23 kb) [file 12889_2015_2563_MOESM1_ESM.docx]

**APPENDIX A**

**Focus Group Survey**

**Review Date (MM/DD/YYYY): [ ][ ]/[ ] [ ]/[ ][ ][ ][ ]**

**Study Code: [ ][ ][ ]**

| Please indicate how much you agree or disagree with each statement. | | | | |
| --- | --- | --- | --- | --- |
| **1 Strongly Agree** | **2 Somewhat Agree** | **3 Somewhat Disagree** | | **4**  **Strongly Disagree** |
| 1. I believe there are enough laws to control environmental risks. | | | [ ] | |
| 1. When there is a really serious health problem, the government will do something about it. Until they tell me about a specific problem, I don’t have to worry. | | | [ ] | |

| Please indicate how much you agree or disagree with each statement. | | | | |
| --- | --- | --- | --- | --- |
| **1 Strongly Agree** | **2 Somewhat Agree** | **3 Somewhat Disagree** | | **4**  **Strongly Disagree** |
| 1. If people work together, they can change the environment. | | | [ ] | |
| 1. There are things I can do that will make a difference in improving the environment. | | | [ ] | |
| 1. Nothing can be done about environmental problems like hazardous waste and air pollution. | | | [ ] | |

| Please indicate how much you agree or disagree with each statement. | | | | |
| --- | --- | --- | --- | --- |
| **1 Strongly Agree** | **2 Somewhat Agree** | **3 Somewhat Disagree** | | **4**  **Strongly Disagree** |
| 1. Most chemicals cause cancer. | | | [ ] | |
| 1. The risk of getting cancer from things like smoking and diet is much greater than the risk of cancer from chemicals in the environment. | | | [ ] | |
| 1. People can protect themselves against health risks from pollution by improving their individual lifestyle, such as by exercising and eating properly. | | | [ ] | |
| 1. If even a tiny amount of a substance that could make me sick were found in my tap water, I wouldn’t drink it. | | | [ ] | |
| 1. I don’t worry about chemicals because there are just too many other things in my life I have to deal with. | | | [ ] | |
| 1. I feel I have very little control over risks to my health. | | | [ ] | |

| **Please indicate how much information you receive from the following sources about your environment.** | |
| --- | --- |
| 1. Television   Almost none 0  A little 1  A fair amount 2  A lot 3 | [ ] |
| 1. Newspaper   Almost none 0  A little 1  A fair amount 2  A lot 3 | [ ] |
| 1. Radio   Almost none 0  A little 1  A fair amount 2  A lot 3 | [ ] |
| 1. Private industry, such as Waste Management or Ford Motor Company   Almost none 0  A little 1  A fair amount 2  A lot 3 | [ ] |
| 1. Chicago City Health Department   Almost none 0  A little 1  A fair amount 2  A lot 3 | [ ] |
| 1. Illinois Department of Health   Almost none 0  A little 1  A fair amount 2  A lot 3 | [ ] |
| 1. Chicago Housing Authority (CHA)   Almost none 0  A little 1  A fair amount 2  A lot 3 | [ ] |
| 1. Environmental Protection Agency (EPA)   Almost none 0  A little 1  A fair amount 2  A lot 3 | [ ] |
| 1. University/College scientists   Almost none 0  A little 1  A fair amount 2  A lot 3 | [ ] |
| 1. Friends and relatives   Almost none 0  A little 1  A fair amount 2  A lot 3 | [ ] |
| 1. People for Community Recovery (PCR)   Almost none 0  A little 1  A fair amount 2  A lot 3 | [ ] |
| 1. Your doctor   Almost none 0  A little 1  A fair amount 2  A lot 3 | [ ] |

| **Please indicate what level of risk the following items are to you and your family, and your community.**  **[Perceived severity]** | | | | | | |
| --- | --- | --- | --- | --- | --- | --- |
| **1**  **Almost no risk** | **2**  **Low risk** | **3**  **Somewhat of a risk** | | **4**  **High risk** | | **5**  **Very high risk** |
|  | | | **Yourself and Family** | | **Your community** | |
| 1. Pesticides in food | | | [ ] | | [ ] | |
| 1. Indoor air pollution | | | [ ] | | [ ] | |
| 1. Depletion of the ozone layer | | | [ ] | | [ ] | |
| 1. Asbestos | | | [ ] | | [ ] | |
| 1. Bacteria in food | | | [ ] | | [ ] | |
| 1. Sewage | | | [ ] | | [ ] | |
| 1. High tension wires | | | [ ] | | [ ] | |
| 1. Waste incinerators | | | [ ] | | [ ] | |
| 1. Global warming | | | [ ] | | [ ] | |
| 1. Sun exposure | | | [ ] | | [ ] | |
| 1. Chemicals | | | [ ] | | [ ] | |
| 1. Outdoor air pollution | | | [ ] | | [ ] | |
| 1. Car accidents | | | [ ] | | [ ] | |
| 1. Drinking water | | | [ ] | | [ ] | |
| 1. Dumping hazardous waste | | | [ ] | | [ ] | |
| **1**  **Almost no risk** | **2**  **Low risk** | **3**  **Somewhat of a risk** | | **4**  **High risk** | | **5**  **Very high risk** |
|  | | | **Yourself and**  **Family** | | **Your community** | |
| 1. Smoking | | | [ ] | | [ ] | |
| 1. Crime | | | [ ] | | [ ] | |
| 1. HIV/AIDS | | | [ ] | | [ ] | |
| 1. Drugs | | | [ ] | | [ ] | |
| 1. Police brutality | | | [ ] | | [ ] | |
| 1. Landfills | | | [ ] | | [ ] | |
| 1. Lead | | | [ ] | | [ ] | |

| **The last set of questions asks general information about you and your background.** | | | |  |
| --- | --- | --- | --- | --- |
| 1. In general, would you say your health is   Excellent 1 Very good 2  Good 3 Fair 4  Poor 5 | | [ ] | |  |
| 47. Gender:  Male 0 Female 1 | | | [ ] | |
| 48. Year of birth: (YYYY) | [ ] [ ] [ ] [ ] | | | |
| 1. What ethnic background best describes you?   African American or Black 1  Asian/Pacific Islander 2  Hispanic/Latino 3  Native American/Alaskan Native 4  White, non-Hispanic 5  Other (SPECIFY: ______________________________) 6  Don’t know/Don’t remember/Refuse to answer 9 | | | [ ] | |
| 1. Are you Hispanic or Latino?   No 0 Yes 1 | | | [ ] | |
| 1. Which of the following best describes your current situation? Are you…   Married 1 Divorced 2 Widowed 3 Separated 4  Never been married 5 Living with a significant other or partner 6 Refused 9 | | | [ ] | |
| 1. What is the highest grade or year of school you completed?   Never attended school 00 Enter grade (up to 11^th^ grade) number High school graduate or GED 12  College 1 year to 3 years (Some college or tech school) 13  College 4 years or more (College graduate) 14 Refused 99 | | | [ ][ ] | |
| 1. Which of the following best describes your work situation?   Employed full-time 01 Employed part-time 02 Out of work for more than 1 year 03 Out of work for less than 1 year 04 Homemaker 05 Student 06 Retired 07  Unable to work or disabled 08  Other (SPECIFY: ______________________________) 77 Refused 99 | | | [ ][ ] | |
